# Finite Element Driven Design Domain Identification of a Beating Left Ventricular Simulator

**DOI:** 10.3390/bioengineering6030083

**Published:** 2019-09-13

**Authors:** Utku Gulbulak, Atila Ertas

**Affiliations:** Department of Mechanical Engineering, Texas Tech University, Lubbock, TX 79409, USA; utku.gulbulak@ttu.edu

**Keywords:** finite element model, left ventricular simulator, left ventricular assist devices, parametric study

## Abstract

Almost ten percent of the American population have heart diseases. Since the number of available heart donors is not promising, left ventricular assist devices are implemented as bridge therapies. Development of the assist devices benefits from both in-vivo animal and in-vitro mock circulation studies. Representation of the heart is a crucial part of the mock circulation setups. Recently, a beating left ventricular simulator with latex rubber and helically oriented McKibben actuators has been proposed. The simulator was able to mimic heart wall motion, however, flow rate was reported to be limited to 2 liters per minute. This study offers a finite element driven design domain identification to identify the combination of wall thickness, number of actuators, and the orientation angle that results in better deformation. A nonlinear finite element model of the simulator was developed and validated. Design domain was constructed with 150 finite element models, each with varying wall thickness and number of actuators with varying orientation angles. Results showed that the combination of 4 mm wall thickness and 8 actuators with 90 degrees orientation performed best in the design domain.

## 1. Introduction

Approximately 92.1 million American adults have at least one type of cardiovascular disease [1]. One third of adults with existing cardiovascular conditions suffer from heart diseases. For the last century, cardiovascular diseases have been considered as the main cause of death in the United States. Depending on the severity and type of the condition, treatment or countermeasure options may vary. Altering lifestyle habits, such as weight control, increasing physical activity, quitting consumption of tobacco products, and moderation of alcohol intake was shown to be beneficial to improve cardiovascular health [2]. For people who have been diagnosed with severe conditions, lifestyle changes and assistive medication may not be enough. Heart transplantation can be considered as the destination therapies in such cases. Yet, the ratio of available heart donors to people on the transplantation waitlist was reported to be not promising [3,4]. 

Left ventricular assist devices (LVADs) are utilized as bridge therapies [5]. LVADs are helpful to patients before transplant surgeries by supporting the circulatory system with an increase in cardiac output. LVADs are classified according to the type of pump they have: pulsatile, axial continuous and centrifugal continuous. The Thoratec Impantable Ventricular Assist Device (IVAD) has a pulsatile flow pump, which can be implanted and employed as right, left, and biventricular support [6]. One advantage of the Thoratec IVAD is its small size. In addition, Abiomed BVS 5000 provides left ventricular support with pulsatile flow pump [7]. The axial flow pumps are utilized in the HeartMate II and the InCor LVAD [8,9]. The InCor LVAD is designed with magnetically suspended bearings to minimize the wear and noise. The EvaHeart provides continuous flow with a centrifugal blood pump [10]. Its design includes an open vane impeller, which results in no blood stagnation. Moreover, the HeartWare Ventricular Assist Device (VAD) has a centrifugal blood pump with a suspended wide-blade impeller, which improves the durability of the device [11]. The VentrAssist has a centrifugal blood pump that is driven by a hydrodynamically suspended impeller [12]. Its design does not include neither shaft nor seals. Therefore, there are no flow stagnations. Development stages of the LVADs benefit from both in-vivo animal studies and in-vitro mock circulation loops (MCLs). Choice of the animal model depends on the device and the desired pathological conditions. Animal models cover bovine, canine, caprine, porcine, and ovine [13,14]. All of the large animal models have their own advantages and disadvantages, however, an ideal animal model that can represent the human cardiovascular system is nonexistent [15]. 

MCLs are considered beneficial since they can reduce the need for in-vivo studies [16]. MCLs consist of compliance chambers, resistance valves, artificial heart valves or flip valves, and a cardiac simulator [17]. Pneumatically driven chambers have been employed, as a cardiac simulator in MCL setups [18,19,20]. However, the shape of these chambers was either cylinder or rectangular prism instead of the native shape of the ventricles. Pantalos et al. [21] conducted a study to assess whether a mock left ventricle can accurately regenerate normal, heart failure, and partial cardiac recovery. The mock left ventricle was modeled as a hemiellipsoid polyurethane sac to mimic the shape of the native left ventricle. The ventricular motion was obtained by placing the mock left ventricle inside a pressure chamber and changing the inside pressure. It was shown that the mock ventricle was able to exhibit Starling characteristics of the natural heart for all the test conditions of the study. Gregory et al. [22] investigated the use of a silicone mock ventricle modeled from a patient-specific failing heart. A three-dimensional model of the mock ventricle was regenerated by CT scanning. Scanned geometry was converted to a mold and the mold cavity was filled with silicone. The silicone ventricle was actuated with a pressure chamber. Results showed that the silicone ventricle was able to accurately simulate the heart failure case. Additionally, it was noted that this approach could be useful to optimize LVAD placement by visualizing inflow. Roche et al. [23] developed a left ventricular (LV) simulator modeled from a generalized left ventricle geometry. The simulator wall was manufactured with silicone. McKibben actuators were chosen as the actuation mode for the simulator and placed inside the simulator wall to mimic the orientation of cardiac muscles. The simulator successfully exhibited apical twist of the left ventricle. However, the performance of the simulator was not investigated with an MCL setup. Baturalp [24] conducted a study to evaluate the performance of a flexible beating LV simulator. Ventricular geometry was designed from mean dimensions of the native ventricle. The simulator wall was made from latex rubber with 1 mm wall thickness and actuated with 4 McKibben actuators with 90 degrees orientation around the wall. It was shown that the simulator was able to exhibit Starling characteristics, yet it provided only 2 liters per minute. Even though the simulator did not provide clinical flow rate of 5 liters per minute, Baturalp’s approach has shown promise by mimicking heart wall motion. However, flow rate should be increased to clinical values, so that it can be considered as a pump in MCL setups. Therefore, there is a need for a parametric study approach to identify a better configuration for the beating LV simulator and achieve higher flow rates by increasing the deformation of the simulator.

To satisfy this need, this study offers a finite element (FE) driven design domain identification of the beating LV simulator that identifies the combination of wall thickness and the number of actuators with orientation angle that results in better deformation. First, a nonlinear FE model of the beating LV simulator was developed. The developed model was validated with a prototype by comparing directional deformation of the apex, rotation of the apex, and the surface deformations. Deformation behavior was considered in dry conditions, without fluid inside, both in the model and the prototype. Secondly, 150 FE models were prepared, each with different wall thickness and the number of actuators with the orientation angles. Wall thickness differed between 1 mm to 5 mm, the number of actuators differed between 4 to 8, and the orientation angle differed between 15 degrees to 90 degrees with increment of 15 degrees. Finally, the design domain was constructed with the directional deformation of the apex and rotation of the apex results from the models. Results showed that the beating LV simulator combination of 4 mm thickness and 8 actuators with 90 degrees orientation had better deformation in the design domain.

## 2. Materials and Methods

### 2.1. Tensile Testing of Latex Rubber

**Figure 1:**
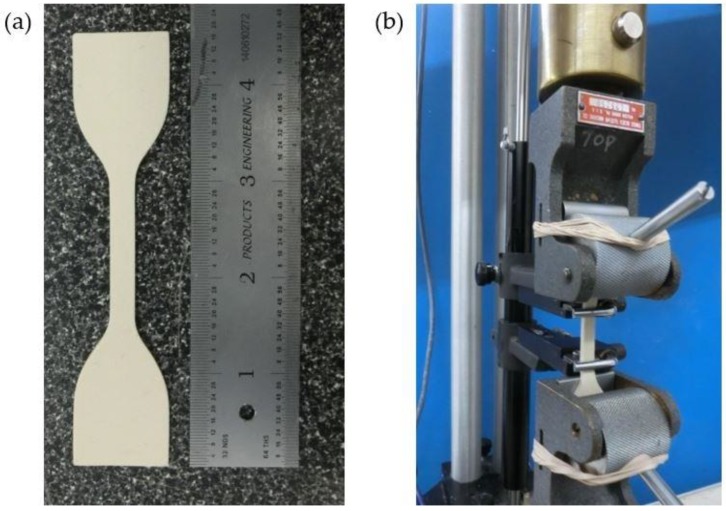
(**a**) Dumbbell test sample. The surface of the sample was smooth and there were no discontinuities on the surface; (**b**) Test sample was clamped at both ends and subjected to a controlled tension until rupture.

Representation of the stress-strain behavior of the latex rubber (AeroMarine Products Inc, San Diego, CA, USA) was important for accurate modeling in FE analysis. The complete stress-strain region was determined with the tensile test (Figure 1). A 185 mm to 130 mm rectangular mold was 3D printed with polylactic acid (PLA). Depth of the mold was 3 mm. The mold was filled with latex rubber and tilted to eliminate air bubbles in the latex. Excess latex rubber was removed with an acrylic roller to obtain a flat surface. Latex rubber was allowed to cure 72 h at room temperature and easily peeled out of the mold. Three test samples were cut out of the latex sheet with die C of ASTM D412-16 [25]. The tensile test was performed for three samples individually. Each test was completed until rupture at the reduced section.

### 2.2. Beating LV Simulator Geometry and Mold Generation

**Figure 2:**
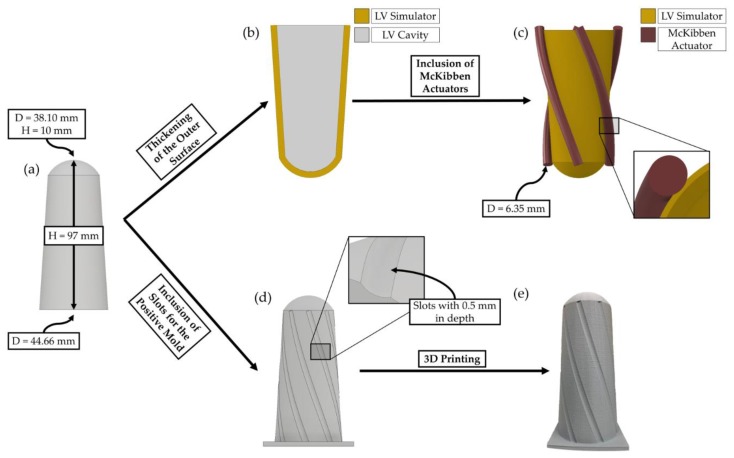
(**a**) Revolved solid body of the left ventricular (LV) cavity. The total height of the cavity is 97 mm. Apex diameter is 38.10 mm and the height of the apex is 10 mm. The diameter of the bottom surface is 44.66 mm; (**b**) Thicken feature in Autodesk Inventor was used to generate desired thickness; (**c**) Diameter of the McKibben actuators is 6.35 mm. The 90 degrees orientation model is given as an example. The crossectional view shows the continuous contact region between the simulator and the actuator; (**d**) 0.5 mm slots were added on the mold body. The 90 degrees orientation mold is given as an example; (**e**) 3D printed 90 degrees orientation mold.

The beating LV simulator geometry and mold were generated in Autodesk Inventor and dimensions were based on Baturalp’s [24] simulator. First, a two-dimensional sketch was prepared and revolved around the vertical axis to create a solid body that represents the LV cavity (Figure 2a). The outer surface of the LV cavity was thickened with the desired thickness (Figure 2b). The helical orientation of the McKibben actuators was defined by the parametric helix equation. Helix coordinates were defined as follows:

X = Rcosθ(1)

Y = Rsinθ(2)

Z = h(3)

where R is the radius of the helix, θ is the orientation angle of actuators, h is the height measured from the origin of the base plane. Helical coordinates for all orientation angles were printed to Excel files by a custom MATLAB script. To achieve a continuous contact region in FE models, there was a 0.5 mm offset between the radius of the helix and radius of the LV simulator at any construction point. A three-dimensional helix sketch was generated with coordinate files. McKibben actuators were modeled as solid cylinders to simplify the FE models [23]. Finally, intersecting regions between the actuators and the simulator were deleted since continuous contact region was needed (Figure 2c). 

A positive mold was generated out of the LV cavity. First, separate sets of coordinate files were generated. Similar to the actuator coordinates, mold coordinates also had an offset to mimic the simulator, therefore slots were created on the mold geometry to provide guides for actuator orientation during manufacturing. A base plate was added to the mold geometry that provides a stable surface during manufacturing (Figure 2d). Finally, generated mold geometry was 3D printed with PLA (Figure 2e).

### 2.3. FE Models

Nonlinear FE models were generated in ANSYS Workbench R19.1. Stress-strain data from the tensile test of the latex rubber was fitted into the five parameter Mooney-Rivlin model [26,27]. The density of the latex rubber was defined as 967.09 kg/m^3^. Roche et al. [23] proposed an efficient way to model McKibben actuators. Mechanical deformation of McKibben actuators was mimicked with thermal deformation by replicating mechanical strains with thermal strains. A McKibben actuator, manufacturing steps are in the following section, was loaded with the air pressure of 550 kPa, working pressure of the simulator, to identify the mechanical strains. The unloaded length and the unloaded diameter of McKibben actuator were 100 mm and 6.35 mm, respectively. The loaded length was measured as 84 mm and loaded diameter was measured as 11.24 mm. Strain along the longitudinal axis, ε_L_, and along the radial axis, ε_R_, were calculated as −16% and 77%, respectively. Mechanical strains were converted to thermal strains as follows:

ε_L_= (ε_thermal_)^L^ = α_L_ ∆T (4)

ε_R_= (ε_thermal_)^R^ = α_R_ ∆T (5)

where α is the thermal expansion coefficient, ∆T is the temperature change, which was arbitrarily determined to be 1000 degrees Celsius. Thermal expansion coefficients in all three directions were derived from Equation (4) and Equation (5) and defined as orthotropic secant thermal expansion coefficients. Young’s Modulus and Poisson’s ratio of McKibben actuators were defined as 1.34 MPa and 0.35 respectively [28]. Density was defined as 4.5 × 10^−10^ g/cm^3^.

**Figure 3:**
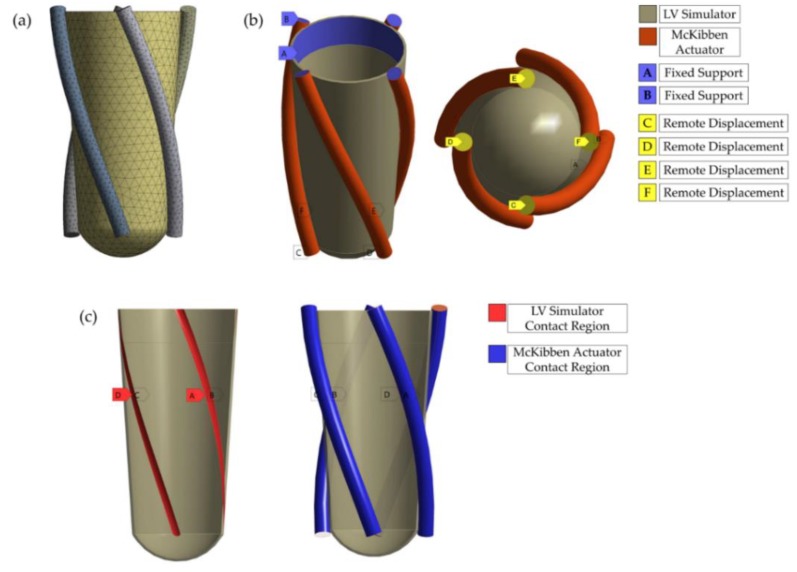
The 90 degrees orientation model is given as an example; (**a**) Meshed geometry with tetrahedral elements; (**b**) Fixed supports were defined on the LV simulator and the McKibben actuators. Remote displacements were added to the bottom surfaces of the actuators with rigid behavior; (**c**) Continuous contact region was defined on the surface of the slot of LV simulator and the surface of the actuators.

LV simulator and McKibben actuators meshed with quadratic tetrahedron elements. Mesh seed sizes were 3.75 mm for LV simulator and 2 mm for actuators (Figure 3a). LV simulator was connected to the artificial heart valve holder in the validation experiment. The inner face of the simulator was fixed onto the outer face of the holder by the top 13 mm. Therefore, the top 13 mm of the inner face of the LV simulator was defined as fixed support (Figure 3b). McKibben actuators were fixed with zip ties and connectors on both ends. To mimic mentioned boundary condition, the top faces of the muscles were considered as fixed supports in the model (Figure 3b). Then, remote displacement type boundary conditions were assigned to the bottom faces (Figure 3b). Bottom faces were free to displace along X, Y and Z axes yet rotation around three axes were not allowed. Moreover, the behavior was chosen as rigid so that the bottom ends of the muscles preserve their shape to satisfy corresponding physical boundary condition. Bonded contact was defined between actuator faces and the LV Simulator (Figure 3c). Lastly, the actuators were assigned to the thermal condition of 1000 degrees Celsius temperature change. A directional displacement probe and a flexible rotation probe were added to the models to generate the design domain. 

### 2.4. Validation of the FE Model

LV simulator with 1 mm wall thickness and 4 actuators with 90 degrees orientation was chosen for the validation of the FE model. 

#### 2.4.1. Prototype Manufacturing

**Figure 4:**
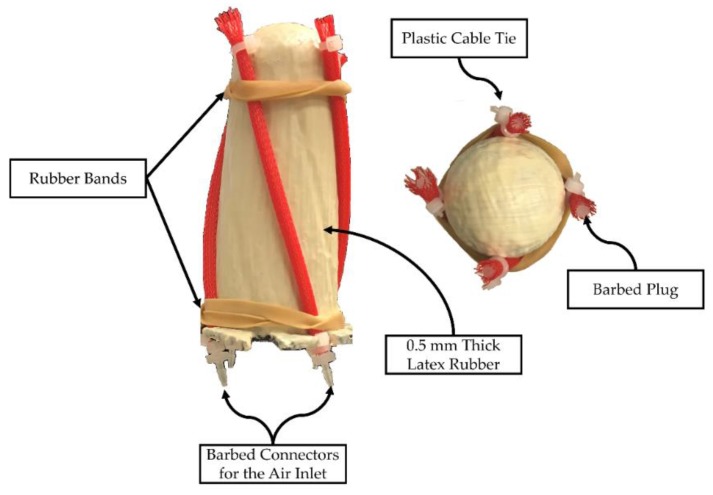
McKibben actuators were placed on the slots. Two rubber bands were added on the bottom and the top to keep actuators in place. Rubber bands were removed after the actuators were fixed on the latex rubber.

In total, 3.175 mm outer diameter latex rubber tubing (McMaster-Carr, 5234K931, Elmhurst, IL, USA) and 6.35 mm diameter expandable polyester sleeving (McMaster-Carr, 9284K12, Elmhurst, IL, USA) were used for McKibben actuators [24]. Length of the tubing was 100 mm. The latex tubes were inserted inside of the sleeving. Whereas one end of the tubes was closed with a barbed plug (5463K73, McMaster-Carr, Elmhurst, IL, USA), a barbed connector (5121K191, McMaster-Carr, Elmhurst, IL, USA) for an air inlet was inserted to the other end. Both ends were reinforced with plastic cable ties (Figure 4). 

Latex rubber was applied on the mold with a 50 mm 100% bristle brush. A thin layer was developed after one application with the brush and was cured by exposure to air. It was observed that 0.5 mm thickness was formed after 4 layers. McKibben actuators were fixed to the LV simulator by applying latex droplets with an RS-2 paintbrush (Figure 4). 4 additional layers were applied and after the 8th layer, LV simulator was allowed to cure for 72 h, then it was peeled off from the mold.

#### 2.4.2. Validation Experiment Setup

**Figure 5:**
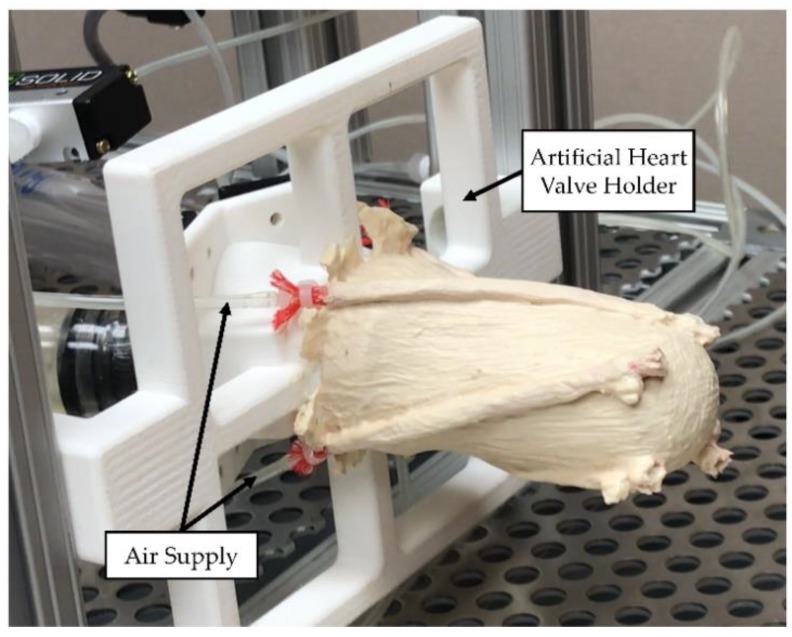
Setup of the validation experiment. All the actuators were loaded simultaneously.

LV simulator was connected to the artificial heart valve holder (Figure 5). Each McKibben actuator was connected to the air source and loaded with 550 kPa. While directional deformation of the apex and the rotation of the apex were measured, surface deformations were observed on the LV simulator.

## 3. Results

### 3.1. Tensile Testing of Latex Rubber

**Figure 6:**
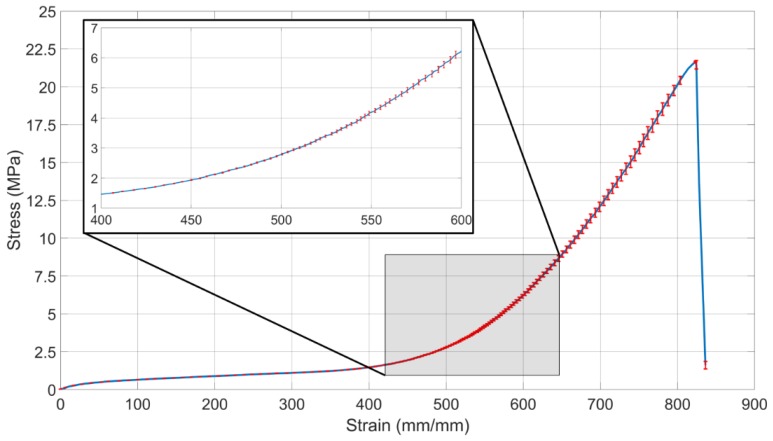
Stress-strain curve of the latex rubber. The blue line indicates the average of three tests and the error bars represent the standard error. The shaded area is magnified to show 400 mm/mm to 600 mm/mm strain range clearly.

Figure 6 shows the stress-strain curve of the latex rubber. The average maximum strain of the test samples was 836 mm/mm before the rupture.

### 3.2. Validation of the FE Model

Directional deformation of the apex and rotation of the apex were selected as the quantitative measures for the validation. Additionally, observed surface deformations were considered as the qualitative measure for the validation. 

**Figure 7:**
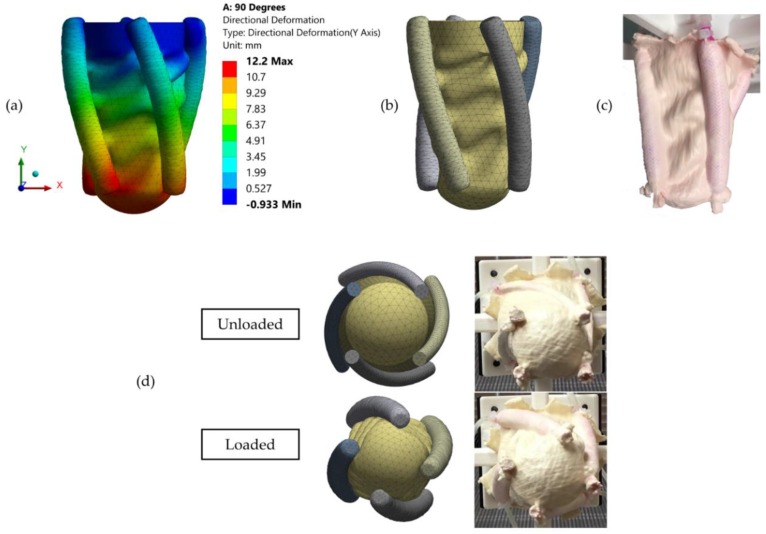
(**a**) Contour bands of the directional deformation along the y-axis. The positive y-axis is located on the upward direction; (**b**) Deformed FE model; (**c**) Deformed LV simulator prototype; (**d**) Before and after loading states of the model and the experiment from the apical view. Similar deformation mechanics were observed on the model and the experiment.

Figure 7a shows contour bands of the directional deformation along the y-axis. Maximum directional deformation was predicted as 12.200 mm and located on the bottom surface of the actuators. In addition, minimum directional deformation was predicted as −0.933 mm and located on the outer surface adjacent to the top surface of the actuators. It was observed that elements were stretched towards the negative y-axis, therefore deformation was predicted as negative. This was also observed in the validation experiment.

While directional deformation of the apex was predicted as 11.029 mm by the FE model, it was measured as 11 ± 0.1 mm on the validation experiment. Experimental measurement matched with FE prediction by 98.82%. Moreover, rotation of the apex was predicted as 37.290 degrees by the FE model and it was measured as 34.670 degrees on the validation experiment. Rotation of the apex on the experiment matched with FE prediction by 92.54%. Surface deformations were observed in both the FE model (Figure 7b) and the validation experiment (Figure 7c) as a result of the folding of the latex rubber. Surface deformations on the FE model were closely matched with the validation experiment. In addition, Figure 7d shows the unloaded and the loaded states for both the model and the experiment. FE model exhibited closely matched behavior with the experiment during loading.

**Figure 8:**
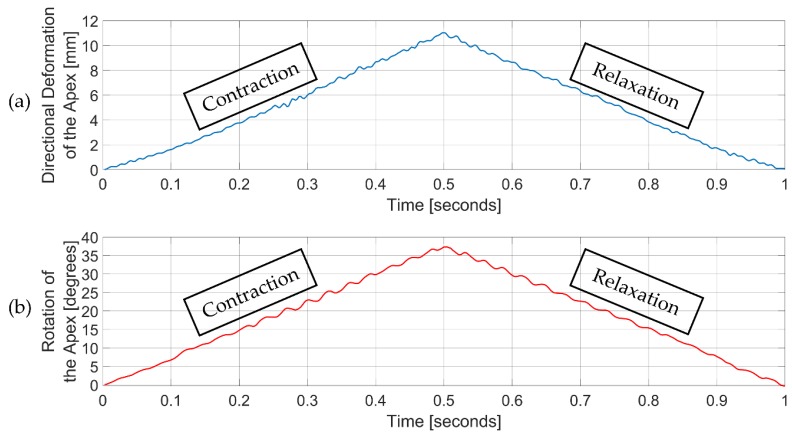
Time change of directional deformation and rotation of the apex over one beating. Actuators were loaded from 0 to 0.5 s and unloaded from 0.5 s to 1 s. (**a**) Directional deformation of the apex; (**b**) Rotation of the apex.

Figure 8 shows the time change of the directional deformation of the apex and the rotation of the apex over one beating from the 1 mm wall thickness and 4 actuators with 90 degrees orientation. Both directional deformation and rotation increases linearly during the contraction of the actuators. Peak values were observed at 0.5 s. Between 0.5 s and 1 s, relaxation of the actuators, directional deformation and rotation decreases linearly.

### 3.3. Design Domain of the LV Simulator

**Figure 9:**
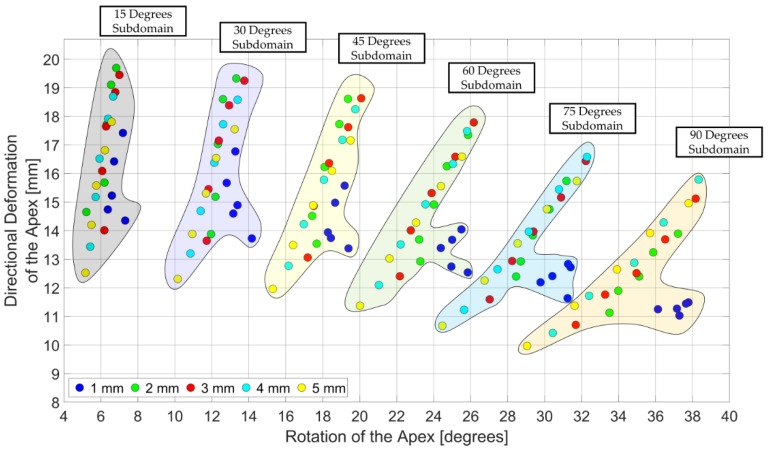
Design domain of the LV simulator. Subdomains were identified with respect to the orientation angle and legend represents the wall thickness of the LV simulator.

After the validation of the FE model. Design domain of the LV simulator was generated with the directional deformation of the apex and the rotation of the apex from FE models (Figure 9). Figure 9 shows that design points with the same orientation angle were located around the same individual regions in the design domain. Therefore, the design domain was divided into six subdomains with respect to the orientation angle.

**Figure 10:**
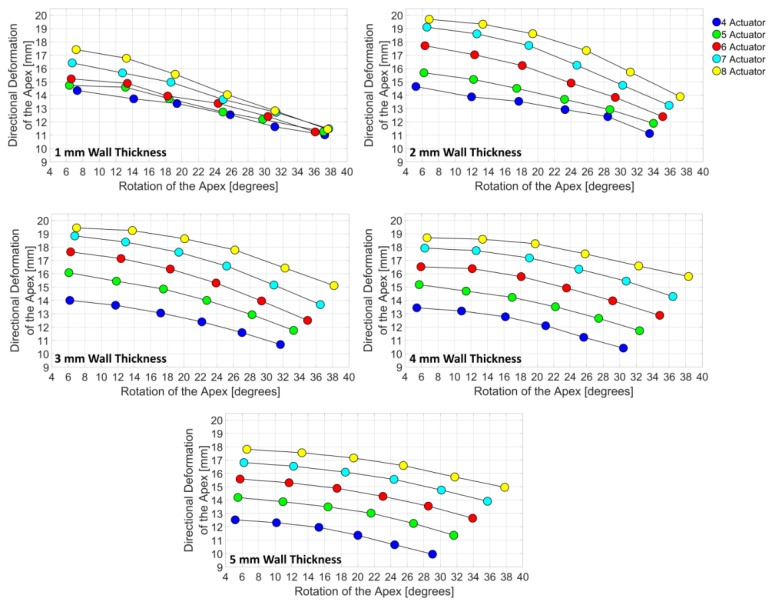
Effect of the number of actuators on different wall thickness in the design domain. Actuator orientation is changing from 15 degrees to 90 degrees, from left to right in the figures.

While 15 degrees subdomain extended between 12 mm to 20 mm and 5 degrees to 8 degrees, 90 degrees subdomain extended between 10 mm to 16 mm and 29 degrees to 39 degrees. Therefore, as the orientation angle increases, deformation behavior, which is a combination of directional deformation and rotation, of the subdomains shifts from directional deformation dominant to rotation dominant. From 4 actuators to 8 actuators, the relationship between the directional deformation and the rotation was shown as linear, for all wall thicknesses in the subdomains except the 1 mm wall thickness. Increase in the number of actuators offered a significant improvement in deformation behavior of the LV simulator for 2 mm, 3 mm, 4 mm and 5 mm models (Figure 10). However, 1 mm models did not benefit from the increase in the number of actuators and showed little or no improvement.

**Figure 11:**
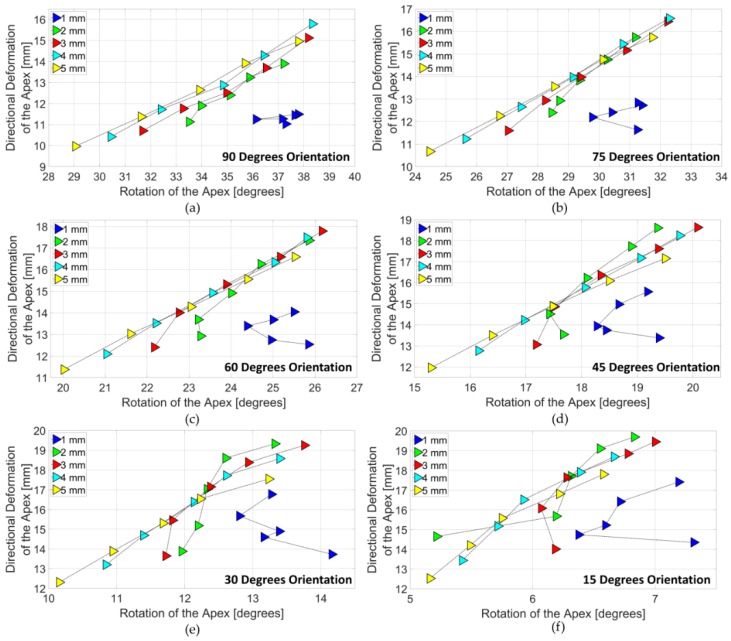
(**a**) 90 degrees subdomain; (**b**) 75 degrees subdomain; (**c**) 60 degrees subdomain; (**d**) 45 degrees subdomain; (**e**) 30 degrees subdomain; (**f**) 15 degrees subdomain.

Figure 11 shows the design subdomains of the LV simulator. In 90 degrees subdomain, as the number of actuator increases from 4 to 8 in 1 mm models, directional deformation of the apex improved only 4% and rotation of the apex improved only 1% (Figure 11a). The improvement of the deformation behavior of the higher wall thicknesses was distinguishable. In 4 mm models, directional deformation of the apex improved as high as 52% In addition, rotation of the apex improved as high as 30% in 5 mm models. 4 mm model with 8 actuators performed best in the 90 degrees subdomain. In the 75 degrees subdomain, directional deformation of the apex improved 10% in 1 mm models as number actuators increased (Figure 11b). However, rotation of the apex did not change. The highest change in directional deformation, 48% improvement, was observed in 4 mm models. Additionally, the highest change in directional deformation occurred in 5 mm models and was 30%. Similar to the 90 degrees subdomain, 4 mm model with 8 actuators performed best in the 75 degrees subdomain. In the 60 degrees subdomain (Figure 11c), while directional deformation of the 1 mm models increased by 12% with 8 actuators, rotation of the 1 mm models decreased by 1%. The decrease in either directional deformation or rotation was only observed in the 1 mm models. Models with 5 mm wall thickness exhibited the highest improvement with 46% and 30% for directional deformation and rotation respectively. Yet, 3 mm model with 8 actuators performed best in the subdomain. 

In the 45 degrees subdomain (Figure 11d), directional deformation of the 1 mm models increased by 16% with the increase in the number of actuators. Rotation decreased by 1%. Highest rotation improvement was found in 5 mm model with 8 actuators. Highest improvement of directional deformation was calculated as 43% and it was found in 3 mm, 4 mm, 5 mm models with 8 actuators. 3 mm model with 8 actuators performed best in the subdomain. In 30 (Figure 11e) and 15 degrees subdomain (Figure 11f), directional deformation increased by 22% and 21%, respectively. The rotation was decreased in both subdomains with 6% and 2%. 5 mm models showed highest improved for not only directional deformation but also rotation. The best directional deformation and the best rotation did not occur with the same wall thickness. In both subdomains, the best directional deformation was observed in 2 mm model with 8 actuators and the best rotation was observed in 3 mm model with 8 actuators. 

**Figure 12:**
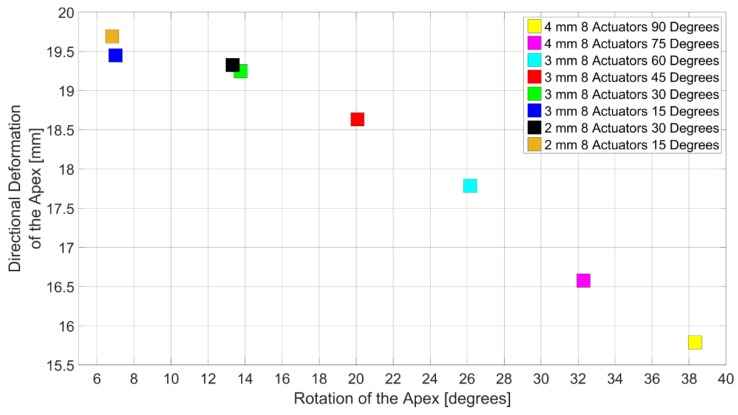
Configurations with the highest directional deformation and the highest rotation in the subdomains.

Figure 12 shows the configurations that exhibited the highest directional deformation and rotation in their subdomains. As expected, 90 and 75 degrees subdomain models exhibited the top two highest rotation among the best configurations. Additionally, 15 and 30 degrees subdomain models exhibited the top four highest directional deformations. The highest directional deformation was 19.69 mm by 2 mm wall thickness and 8 actuators with 15 degrees orientation. Directional deformations of the 90 and 75 degrees subdomains were less than the highest by 22% and 17% respectively. However, their rotation was found to be greater than the 2 mm model by 82% and 79%. In between 90 and 75 degrees subdomains, rotation of the 90 degrees subdomain is greater than the rotation of the 75 degrees subdomain by 20%. In addition, directional deformation of the 75 degrees subdomain is greater than 90 degrees subdomain by 5%. 

## 4. Discussions

Validated FE model had 24,281 quadratic tetrahedral elements and resulted in convergence in 29 min. To investigate the accuracy of the results with a change in the number of elements, a model with 132,199 elements was generated with 2 mm mesh seed size for both LV simulator and McKibben actuators. The model with the high number of elements resulted in convergence in 1 h 45 min. While the directional deformation of the apex in the model with the high number of elements was 10.957 mm, it was 11.029 mm in the validated model, discussed previously. Moreover, rotation of the apex was 37.770 degrees in the model with the high number of elements and was 37.290 degrees in the validated model. Difference between the results of the two models was not significant, yet computation time of the model with the higher number of elements was almost four times greater than the validated model. Therefore, to reduce the total computation time of this study, mesh seed size of 3.75 mm for LV simulator and mesh seed size of 2 mm for McKibben actuators were used. Due to the observed convergence difficulties, mesh seed size of the LV simulator was tuned between 3.5 mm to 3.85 mm. 

Increasing the number of McKibben actuators can be considered as a guaranteed option to improve deformation of the LV simulator since the deformation of the LV simulator is driven by them. Also, wall thickness plays a role in the improvement of deformation. As wall thickness increases, the contact area between the LV simulator and actuators increases. The 1 mm models did not have the extra contact area due to the thickness and more importantly, their deformation was not improved with the higher number of actuators. Therefore, 1 mm wall thickness is not a good practice for the LV simulator configuration. However, it is interesting to note that even though the best performance was achieved with different wall thicknesses in the subdomains, 5 mm models did not exhibit the best performance. It was observed that as the orientation angle increases, best-performing wall thickness was increased up to 4 mm. Almost half of the actual ejection fraction of the human heart is provided by the twisting of the heart [29]. It is expected to observe similar behavior with the LV simulator. Therefore, LV simulator configuration of 4 mm wall thickness and 8 actuators with 90 degrees orientation is chosen as the best in the design domain with the highest rotation. 

In the future study, the developed FE model will be extended to a fluid-structure interaction (FSI). The first aim of the future study is to predict the ejection fraction of the LV simulator and developed model will be validated with an experiment. Prediction of the ejection fraction will help to further identify the effect of wall thickness, the number of actuators and actuator orientation. Furthermore, flow inside the LV simulator will be also investigated and compared with the native intraventricular flow [30,31]. The second aim of the future study is to predict the filling and ejection mechanics. Artificial heart valve holder and the artificial heart valves will be added to the FSI model to investigate flow through the valves and mechanics of the artificial valves [32,33].

## 5. Conclusions

In this paper, design domain identification of the beating LV simulator was presented. LV simulator was built with latex rubber and helically oriented McKibben actuators. A nonlinear FE model was developed to predict the directional deformation and rotation of the apex. Latex rubber was modeled with the five parameter Mooney-Rivlin model and mechanical strain of the actuators was replicated with thermal strains. Developed FE model was validated with a prototype. Predictions of directional deformation of the apex and rotation of the apex were in good agreement with the ones measured on the validation experiment. Additionally, observed surface deformations on the experiment closely matched the ones on the FE model. Design domain was constructed with 150 FE models of LV simulator configurations, each with different wall thickness, the number of actuators and orientation angles to identify the configuration. which exhibits the best deformation performance in the domain. 

The results showed that the design points of the same orientation angles were located around individual regions. Individual regions were defined as subdomains. Deformation behavior of models changed from directional deformation dominant to rotation dominant as orientation angle increases. The relationship between the directional deformation and the rotation was evaluated in each subdomain. All the models except 1 mm exhibited a linear behavior between two. The 1 mm models were found not to be good practice for LV simulator since their deformation was shown little or no improvement. Best performing configurations were identified in each subdomain. The LV configuration of 4 mm wall thickness and 8 actuators with 90 degrees orientation was identified as the best in the design domain. In the future study, the developed FE model will be used to develop an FSI model to predict the ejection fraction, flow through artificial valves and flow inside the LV simulator.
